# Simultaneous Manifestation of Chronic Myelomonocytic Leukemia and Multiple Myeloma during Treatment by Prednisolone and Eltrombopag for Immune-Mediated Thrombocytopenic Purpura

**DOI:** 10.1155/2016/4342820

**Published:** 2016-08-14

**Authors:** Masao Hagihara, Morihiro Inoue, Kenichiro Kodama, Tomoyuki Uchida, Jian Hua

**Affiliations:** Department of Hematology, Eiju General Hospital, 2-23-16 Higashi-Ueno, Taito-ku, Tokyo 110-8645, Japan

## Abstract

An 80-year-old man was admitted to our hospital because of severe thrombocytopenia. He was diagnosed with idiopathic thrombocytopenia, and prednisolone together with eltrombopag was started, leading to significant improvement of platelet counts. Four years later, there was a prominent increase of peripheral blood monocytes, which was accompanied by recurrence of thrombocytopenia. Bone marrow aspirates and serum electrophoresis revealed coexistence of chronic myelomonocytic leukemia (CMML) and multiple myeloma (MM). The patient received lenalidomide plus dexamethasone therapy but died due to exacerbation of the disorder. It was supposed that thrombocytopenia was secondarily caused by CMML and MM developed at a later period.

## 1. Introduction

Immune-mediated thrombocytopenic purpura (ITP) is a disorder which occurs spontaneously as idiopathic thrombocytopenic purpura or develops secondarily from various disorders, such as infection or* malignancies* through mechanisms of immune alteration [1]. Myelodysplastic syndrome (MDS) is one such underlying disorder that manifests isolated thrombocytopenia, although the incidence is quite low [2]. Chronic myelomonocytic leukemia (CMML) overlaps pathophysiologically with MDS and is categorized as MDS/MPN (myeloproliferative neoplasm) in the WHO 2008 classification.

Patients with multiple myeloma (MM) or monoclonal gammopathy of undetermined significance (MGUS) have a significantly high risk of developing MDS, CMML, or acute myeloid leukemia (AML) compared with healthy controls. Furthermore, there have been several case reports that demonstrated the simultaneous occurrence of MM/MGUS and myeloid malignancy. In contrast, there has been no report of MDS or CMML preceding the development of MM.

In the present case, it was concluded that CMML likely existed as an underlying disorder for the development of ITP, which had been in remission under successful treatment with eltrombopag, a recombinant thrombopoietin receptor (TPO-R). Four years later, CMML exacerbated simultaneously with the appearance of MM.

## 2. Case Report

**Figure 1:**
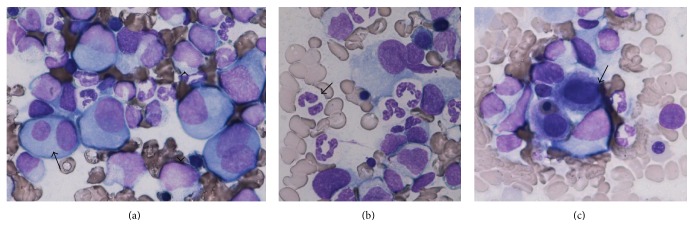
Bone marrow aspirate on second admission (Giemsa staining). A significant increase of monocytes (arrowhead) as well as plasma cells (arrow) was observed in bone marrow aspirates ((a), ×400 in magnification). Myelodysplastic change was also detected, showing as hypogranulated neutrocytes with Pseudo-Pelger nuclei ((b), ×1000 in magnification) or micromegakaryocytes ((c), ×1000 in magnification).

**Figure 2:**
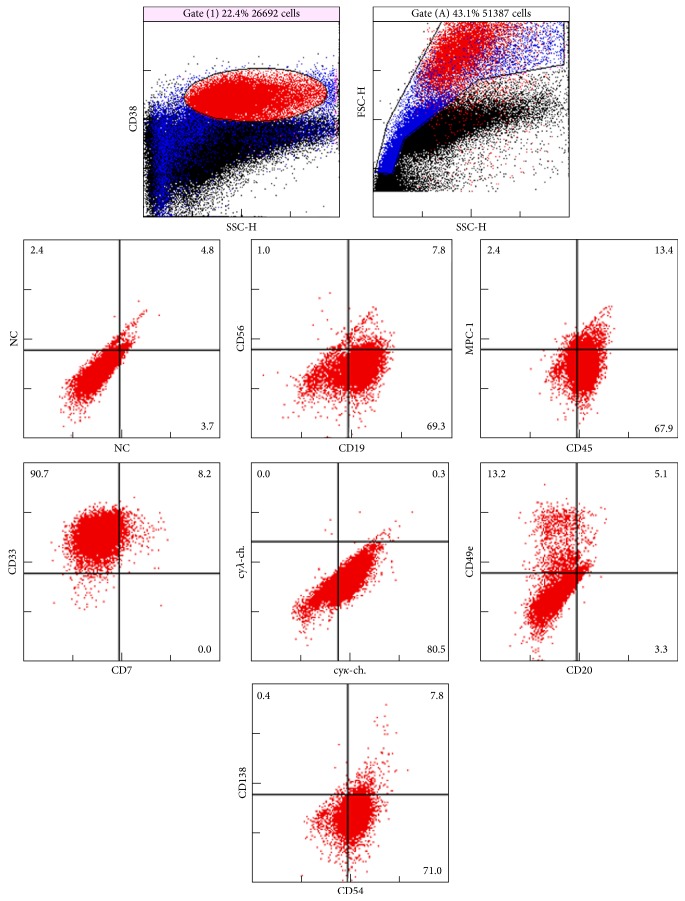
Flow cytometry of bone marrow. CD38 positive cells were positively stained with CD19, CD33, and *κ* antigens and negatively with CD138, CD20, and *λ* antigens.

An 80-year-old man was referred to our hospital because of severe thrombocytopenia (Table 1). Bone marrow (BM) study showed an increased number of megakaryocytes. Neither significant increase of monocytes nor plasma cells were observed. As dysplastic change was* not evident*, he was diagnosed with ITP. At admission, prednisolone was started at 0.5 mg/kg, which did not result in significant improvement of platelet counts (1.7 × 10^4^/mm^3^ to 2.3 × 10^4^/mm^3^) for 1 month, and eltrombopag was added at 12.5 mg/day as a starting dose and then gradually increased to 37.5 mg/day during 4 months. Platelet counts continued to be stable (at around 10 × 10^4^/mm^3^) through this treatment (Table 2). Four years later, there was an increase of peripheral blood monocytes, which was then accompanied by decreased platelet counts and severe anemia (Table 3). At this period, bone marrow examination was again performed and revealed a significant increase in monocytes as well as plasma cells (Figure 1), as determined based on the *κ* chain expression on CD38 gating by flow cytometry (Figure 2). Peripheral blood (PB) monocytes exceeded 1000/mm^3^ and IgG-*κ* typed M-protein was detected by an analysis of serum electrophoresis. Therefore, it was diagnosed that CMML and MM were simultaneously present. As anemia together with deterioration of renal insufficiency was considered to be MM-related symptoms, treatment by lenalidomide plus dexamethasone (starting doses of 25 mg/day and 20 mg/week, resp.) was started on day 1 of a second admission. On day 7, although both PB-monocytes and serum IgG had significantly decreased, the treatment was discontinued because of disseminated intravascular coagulation, possibly due to tumor lysis syndrome. On day 35, the patient was complicated with septic shock. Thereafter, his general condition rapidly worsened and he eventually died due to deterioration of CMML.

## 3. Discussion

It has been recognized that MDS or AML (MDS/AML) can develop in patients with MM [3]. The use of anticancer drugs, especially melphalan, has been considered as the main factor for such elevated risk [4, 5]. Additionally, MM patients have a 2.4-fold to 8.01-fold increased risk of MDS/AML even without such cytotoxic treatment [6, 7]. On the contrary, simultaneous occurrence of MDS/AML and MM is quite a rare event. In the past, 27 cases of such coexistence of plasma cell dysplasia and myeloid malignancy, including 16 cases with MDS, have been reported [8–20]. Only 2 cases were diagnosed as CMML, while 6 out of 8 AML cases were diagnosed as myelomonocytoid type (AML-M4 or AML-M5 in FAB classification).

It has been demonstrated that myeloma cells express multiple hematopoietic markers, including myelomonocytic antigens [21], and occasionally express biphenotypic features not only of myelomonocytic but also of erythroid, megakaryocytic, and T-lymphoid lineages [22]. It was also reported that IgG was produced in supernatants from CD14 positive monocytes in such a CMML/MM case. In addition, a rearranged band of IgH gene from CD14+ peripheral monocytes was identical to that from bone marrow containing plasma cells [8]. According to these findings, it has been speculated that the neoplastic transformation deriving from a common progenitor results in the occurrence of such hybrid hematological disorders. In the present case, myeloma cells expressed CD19 antigen, which generally remains in polyclonal but is absent in monoclonal plasma cells and lacked CD138 antigen, which is commonly expressed in myeloma cells [23]. In addition, CD33 antigen, which is expressed in myelomonocytoid but not in plasma cells, was significantly expressed. From these results, it was highly likely that CMML and myeloma cells in the present case originated from these immature, poorly differentiated progenitors or such atypical antigen expression might simply suggest the malignant, but not reactive (benign), properties of the increasing plasma cells, irrespective of the common progenitors.

At disease onset, neither proliferation of plasma cells nor M-peak by analysis of serum protein fractionation was observed. In contrast, the number of PB-monocytes gradually increased and finally exceeded 1000/mm^3^ during the 4-year period of prednisolone plus eltrombopag treatment. Therefore, it was concluded that CMML existed as an underlying disorder when the patient was firstly diagnosed with ITP, and the present case is the first report of myeloid malignancy proceeding the occurrence of MM.

It has been widely recognized that MDS with CMML is complicated with various types of autoimmune disorders, such as Sweet's disease [2, 24]. It has also been reported that immune-mediated thrombocytopenia can occur in CMML [2, 24]. Hadjadj et al. reported that ITP occurred in the early stage of CMML with no further progression of the disorder. In their report, a favorable response to treatment, including corticosteroid or TPO-R agonist, was obtained. Ineffective platelet production secondary to disordered maturation or proliferation of megakaryocytes has been considered as the other main mechanism of thrombocytopenia in MDS [25]. Eltrombopag has been shown to be useful to treat thrombocytopenia in MDS patients [26]. Platelet counts were improved or remained stable in 9 out of 12 cases, despite azacitidine treatment [27]. A meta-analysis study also demonstrated that romiplostim, another TPO-R agonist, significantly decreases the bleeding or platelet transfusion rate [28]. In the present case, eltrombopag was shown to be effective in maintaining platelet counts for as long as 4 years before diagnosis of CMML/MM.

The safety of long-term treatment by TPO-R agonist has recently been reported and only 2 patients suffered from lymphoid malignancies of diffuse large B-cell and Hodgkin lymphoma [29]. The relative risk of AML progression with romiplostin was 1.36; however, this result was judged as having a high risk of bias [28]. In addition, eltrombopag inhibits proliferation of leukemic cells through a TPO-R independent mechanism in vitro [27, 30, 31]. From these reports, it was concluded that eltrombopag was not involved in either the progression of CMML or the development of MM.

Lenalidomide, an immunomodulatory drug, is widely known as one of the key drugs for initial treatment of MM and also has been approved for low or low-intermediate risk MDS with del(5q) [32, 33]. The drug is also effective, even if to a lesser extent in non-del(5q) MDS. In an MDS-002 trial, the drug demonstrated transfusion independence in one quarter of MDS patients not harboring del(5q) [34]. It also shows a clinical response in CMML patients (25% of overall response), when combined with metronomic melphalan through an antiangiogenic effect [35]. We selected this drug to treat both MM and CMML. However, only a temporal improvement of these disorders was obtained, and eventually, the treatment was discontinued because of decreased performance status due to complications of the severely infectious disease.

Other effective treatments with azacitidine, an analog of pyrimidine nucleoside, have been used in various myeloid malignancies, including CMML [36]. Clinical trials with combination therapy of lenalidomide and azacitidine have been reported. In one study, an overall response rate of 72%, including 44% with complete response, was obtained [37]. Such combination therapy might be a promising option as long as the general conditions of the patient were favorable enough to continue the chemotherapy.

## Figures and Tables

**Table 1:** Laboratory data on first admission.

WBC	3800/mm^3^
Band + seg	15.5%
Baso	0.5%
Eosino	0%
Mono	28%
Lymph	26%
RBC	439 × 10^6^/mm^3^
Hb	13.3 g/dL
MCV	92.7
MCH	30.3
Platelets	3.6 × 10^4^/mm^3^
TP	7.1 g/dL
BUN	13.5 mg/dL
Cr	0.89 mg/dL
Na	140 mEq/L
K	4.7 mEq/L
AST	17 U/L
ALT	12 U/L
LDH	114 U/L
IgG	1729 mg/dL
IgA	404 mg/dL
IgM	46 mg/dL
PAIgG	118 ng/10^7^ cells

PAIgG: platelet-agglutinated IgG.

**Table 2:** The number of PB-monocytes and platelets during 4 years.

	*X*-4 years	*X*-3 years	*X*-2 years	*X*-1 years	*X*-8 months	*X* years
WBC (/mm^3^)	3800	7900	7500	6600	11000	17500
Monocyte (%)	28	24.5	27.5	26.7	32	51.5
Monocytes number (/mm^3^)	1064	1935	2025	1780	3550	8925
Platelet (×10^4^/mm^3^)	3.6	5.3	5.6	6.9	9.2	4.5

**Table 3:** Laboratory data on second admission.

WBC	17500/mm^3^
Blast	0.5%
Myelo	5.5%
Band	4.0%
Seg	32.5%
Mono	51.5%
Lymph	4.5%
RBC	252 × 10^6^/mm^3^
Hb	7.7 g/dL
Platelets	4.5 × 10^4^/mm^3^
TP	7.6 g/dL
Alb	2.9 mg/dL
BUN	64.2 mg/dL
Cr	2.85 mg/dL
UA	16.5 mg/dL
Na	144 mEq/L
K	4.7 mEq/L
Cl	112 mEq/L
Ca	8.1 mEq/L
AST	12 IU/L
ALT	6 IU/L
LDH	248 IU/L
ALP	106 IU/L
IgG	3152 mg/dL
IgA	82 mg/dL
IgM	29 mg/dL
*β*-2MG	12.9 mg/L
Free light chain *κ*	2100 mg/L
Free light chain *λ*	5.8 mg/L
*κ*/*λ* ratio	362
